# Supplementary material for: MetaRibo-Seq measures translation in microbiomes
Source: Nat Commun. 2020 Jun 29;11:3268. doi: 10.1038/s41467-020-17081-z (PMC7324362; doi:10.1038/s41467-020-17081-z)
Supplement: Supplementary file 10 — Supplementary Data 7 [file 41467_2020_17081_MOESM10_ESM.zip › File2/Confidence_High_Taxonomy/158996_out.krona.html]

Javascript must be enabled to view this page.

members
magnitude
magnitudeUnassigned
count
unassigned
taxon
rank

158996\_out

2


SRS015190\_contig\_number\_contig-100\_19798.19798
1

1
superkingdom
2

1239
1
phylum

1
186801
class

1
186802
order

1
186803
family

1
572511
genus


SRS023715\_contig\_number\_contig-100\_8259.97993
40520
1
species
